# Analysis of factors associated with positive surgical margins and the five-year survival rate after prostate cancer resection and predictive modeling

**DOI:** 10.3389/fonc.2024.1360404

**Published:** 2024-06-06

**Authors:** Kai Li, Yantao Zhang, Sinan Tian, Qingguo Su, Yanhui Mei, Wei Shi, Jingyuan Cao, Lijuan Song

**Affiliations:** ^1^ Department of Urology, Binzhou Medical University Hospital, Binzhou, China; ^2^ Department of Anesthesiology, Binzhou Medical University Hospital, Binzhou, China

**Keywords:** prostate cancer, radical prostatectomy (RP), survival time, logistic model, receiver operating characteristic curve (ROC curve)

## Abstract

**Background:**

This study analyzed the risk factors associated with positive surgical margins (PSM) and five-year survival after prostate cancer resection to construct a positive margin prediction model.

**Methods:**

We retrospectively analyzed the clinical data of 148 patients treated with prostatectomy. The patients were divided into PSM group and Negative surgical margins (NSM) group. Several parameters were compared between the groups. All patients were followed up for 60 months. The risk factors for PSM and five-year survival were evaluated by univariate analysis, followed by multifactorial dichotomous logistic regression analysis. Finally, ROC curves were plotted for the risk factors to establish a predictive model for PSM after prostate cancer resection.

**Results:**

(1) Serum PSA, percentage of positive puncture stitches, clinical stage, surgical approach, Gleason score on puncture biopsy, and perineural invasion were significantly associated with the risk of PSM (P < 0.05). Serum PSA, perineural invasion, Gleason score on puncture biopsy, and percentage of positive puncture stitches were independent risk factors for PSM. (2) Total prostate-specific antigen (tPSA) by puncture, nutritional status, lymph node metastasis, bone metastasis, and seminal vesicle invasion may be risk factors for five-year survival. Lymph node metastasis and nutritional status were the main risk factors for the five-year survival of patients with prostate cancer. (3) After plotting the ROC curve, the area under the curve (AUC) [AUC: 0.776, 95%, confidence interval (CI): 0.725 to 0.854] was found to be a valid predictor of PSM; the AUC [AUC: 0.664, 95%, confidence interval (CI): 0.576 to 0.753] was also a valid predictor of five-year survival (P < 0.05). (4) The scoring system had a standard error of 0.02 and a cut-off value of 6. It predicted PSM after prostate cancer resection with moderate efficacy.

**Conclusions:**

Serum PSA, perineural invasion, puncture biopsy Gleason score, and percentage of positive puncture stitches were independent risk factors for positive surgical margins (PSM). Also, lymph node metastasis and nutritional status were the main risk factors for the five-year survival of patients with prostate cancer. Overall, the prediction efficacy of this scoring system concerning the risk of PSM after prostate cancer resection was moderate.

## 1 Introduction

Prostate cancer commonly occurs in the urinary system of males. It has the highest incidence rate among different types of cancer in males in the US and Europe (1–3). Prostate cancer has no specific symptoms. Although awareness among people and screening for prostate-specific antigen (PSA) by healthcare institutions has increased in China, the rate of incidence of new prostate cancer cases has increased significantly in recent years. Thus, in the Chinese population, prostate cancer has become a serious health concern (4, 5). According to the Global Cancer Data Center study, the five-year survival rate of patients with early-stage prostate cancer is 100%, whereas, the five-year survival rate of patients with late-stage prostate cancer is only 28% (6). As the disease progresses, patients often develop lower urinary tract symptoms (LUTS), including straining to urinate, dribbling, frequency, urgency, difficulty urinating, increased nocturia, hematuria, etc. The most common methods used to diagnose prostate cancer in clinical settings include serum prostate-specific antigen (PSA) assessment, rectal finger examination (RFE) and transrectal ultrasound (TRUS) examination, pelvic MRI, CT scanning, and isotope bone scanning. However, the gold standard for diagnosis is prostate puncture biopsy (7, 8). The combination of serum PSA levels, clinical staging, and Gleason score of puncture pathology can be used to classify clinically limited prostate cancer into low-risk, intermediate-risk, and high-risk groups. This classification system can help decide the treatment regimen for patients with prostate cancer.

The best treatment modality for prostate cancer patients is determined by assessing their Gleason score, serum PSA levels, clinical stage, life expectancy, and self-reported general condition, along with the presence of distant metastases (9). Commonly used clinical treatments include close monitoring and active surveillance, resective prostatectomy, external radiation therapy, proton therapy, cryoablation, high-energy focused ultrasound therapy, and endocrine therapy (10). Prostatectomy is the most commonly used method for treating prostate cancer. It is the most effective treatment strategy for limited prostate cancer and some types of high-risk prostate cancer (11). After undergoing excisional prostatectomy, patients have a five-year disease-specific survival rate of >95% (12). Laparoscopic prostatectomy is the gold standard for treating limited prostate cancer. However, even after undergoing extremely successful intraoperative treatment, 10–40% of patients present with PSM pathology after prostate cancer resection (13).

PSM refers to the presence of cancer cells on the ink-stained surface of the prostatectomy specimen. Surgical PSM can be of two types. In the first type, the cancerous tissue invades outside the envelope, leaving cancerous tissue outside the prostate on the ink-stained margins, i.e., true positive. In the second type, the cancerous tissue is confined to the envelope. In such a case, if the periprostatic fascia and envelope are cut and accidentally enter the prostate gland, a portion of the fascia and envelope disappears from the specimen, leaving cancerous tissue inside the prostate on the ink-stained margins, i.e., false positive (14). Men with post-prostate cancer resection specimens suggestive of PSM have a higher chance of experiencing clinical biochemical recurrence (15). A large multi-institutional study showed a 3.7-fold association between PSM and the risk of biochemical recurrence in nearly 6,000 men treated with prostate cancer resection. Spahn et al. analyzed more than 7,000 patients who underwent prostate cancer resection. They constructed a multivariate model and included factors such as age, Gleason score, PSA, extraperitoneal invasion, and seminal vesicle invasion and found that PSM was independently associated with biochemical recurrence (HR 2.3, 95% CI 2.1–2.6, p < 0.001) (16). Therefore, achieving negative surgical margins (NSM) is crucial to increase the chances of survival among prostate cancer patients. However, the proportion of patients with locally progressive intermediate-risk and high-risk prostate cancer undergoing prostatectomy has recently increased considerably (17), which in turn has significantly increased the risk of postoperative PSM. Several studies have shown that postoperative PSM in prostate cancer is correlated with post-penetration Gleason score, PSA level, tumor infiltration in seminal vesicles, clinical stage of the tumor, size (volume) of the prostate, proficiency of the surgeon, the age and body mass index of the patient, and biopsy nerve infiltration (18–21).

Although many studies have investigated PSM in prostate cancer, most researchers conducted cross-sectional surveys in prostate cancer patients. These researches have shown that serum PSA, perineural invasion, puncture biopsy Gleason score, and percentage of positive puncture stitches were independent risk factors for PSM. Moreover, lymph node metastasis and nutritional status were found to be the main risk factors for the five-year survival of patients with prostate cancer. This scoring system has moderate efficacy in predicting the risk of PSM after prostate cancer resection.

## 2 Methods

### 2.1 General information

We retrospectively analyzed the clinical data of 148 patients who underwent prostatectomy from January 2012 to December 2016 in the Department of Urology, Binzhou Medical University Hospital. As this was a case-control study, the postoperative specimens were divided into an NSM group (negative surgical margins group, n = 72) and a PSM group (positive surgical margins, n = 76), according to their postoperative margin status. The patients were followed up for 60 months. The inclusion criteria were as follows: (i) patients diagnosed with prostate cancer by transrectal ultrasound-guided prostate aspiration biopsy; (ii) patients who underwent prostate cancer resection, including open prostate cancer resection, laparoscopic prostate cancer resection, and robot-assisted laparoscopic prostate cancer resection, in our hospital. The exclusion criteria were as follows: (i) patients who underwent neoadjuvant therapy, radiotherapy, chemotherapy, or electrodesiccation of the prostate before prostate cancer resection; (ii) cases with missing raw data; (iii) patients with metastatic lesions were excluded by chest CT or X-ray, pelvic MRI or CT, and whole-body bone scan (**Supplementary Figure S1**); (iv) patients with a preoperative puncture biopsy of <13 stitches; (v) patients with postoperative pathology indicating prostatic hyperplasia or prostatic intraepithelial neoplasia. The study was reviewed and approved by the Ethics Committee of the Binzhou Medical University Hospital (LW-99). All patients and their family members provided informed consent.

### 2.2 Univariate and logistic regression analysis

PSM univariate and logistic regression analysis: The clinical data collected included information on age, serum PSA, Gleason score of puncture biopsy tissue, percentage of positive puncture biopsy stitches, presence of perineural invasion of puncture biopsy tissue, prostate volume, clinical stage, and surgical procedure in both groups of patients. The clinical data of the two groups were compared, and the risk factors for PSM were determined by conducting a univariate analysis and multifactorial dichotomous logistic regression analysis, in which factors with a P-value below 0.1 were included.

Univariate and logistic regression analysis of five-year survival: The clinical data collected included information on age, duration of surgery, intraoperative bleeding, number of postoperative PSM cases, postoperative serum PSA, rectal injury cases, urinary incontinence cases, and anastomotic urethral fistula cases for both groups of patients. Postoperative serum PSA represented serum PSA levels that were assessed one year after surgery. The data on urinary incontinence was recorded one year after surgery. Data on erectile dysfunction indicated the absence of erectile dysfunction before surgery and the inability to consistently obtain or maintain an erection sufficient for satisfactory sexual intercourse after surgery. The clinical data of the two groups were initially compared by one-way analysis. Based on the results of this analysis, factors with a P-value below 0.1 were included in a multifactorial dichotomous logistic regression analysis to identify the risk factors for five-year survival.

### 2.3 PSM prediction model

Prediction modeling: Each risk factor score was assigned based on the dominance ratio (OR) value of each risk factor for a PSM, and the sum of the risk factor scores was recorded as the total score for that patient.

Evaluation method: The diagnostic effectiveness was evaluated based on the area under the receiver operating characteristics (ROC) curve. When the area under the curve was >0.9, 0.7–0.9, and 0.5–0.7, the diagnostic effectiveness was considered to be high, medium, and low, respectively.

Predictive model application: We scored 50 prostate cancer patients who were admitted between January 2021 and January 2022 using the scoring system to assess its effectiveness.

### 2.4 Statistical analysis

All statistical analyses were performed using the SPSS 20.0 software (IBM Corporation, Armonk, NY, USA). The patients were divided into the PSM group and the NSM group. The correlation factors between the two groups were compared by performing the *X^2^* test. The common factor variables that were non-normally distributed were analyzed, and the results were presented as the mean ± standard deviation (M ± SD) unless otherwise stated. Count data were expressed as percentages (%), and the differences in count data between the groups were determined by Chi-square tests. Logistic regression analysis was performed to analyze the risk factors for patients with PSM and survival time. Finally, ROC curves were plotted, and the area under the curve (AUC) was calculated. All differences between groups were considered to be statistically significant at p < 0.05.

## 3 Results

### 3.1 Analysis of the risk factors for PSM

The results of the univariate analysis showed significant differences in serum PSA, Gleason score in the puncture biopsy group, percentage of positive puncture biopsy stitches, presence of perineural invasion in the puncture biopsy tissue, clinical stage, National Comprehensive Cancer Network (NCCN) risk classification, and surgical approach between the two groups (P < 0.05). However, the differences in age and prostate volume between the two groups were not significant (P > 0.05) (**Table 1**).

**Table 1:** Comparison of one-way analysis of each risk factor in the PSM/NSM group.

Variables	PSM group (n=76)	NSM group (n=72)	*X^2^*	P value
PSA (ng/mL)			10.398	0.025
<10	4	15		
10-20	17	23		
≥20	55	34		
Percentage of positive punctures			43.17	<0.001
<25%	12	31		
25-49%	16	20		
≥50%	48	21		
Puncture Gleason score			37.73	<0.001
≤6	23	43		
7	16	14		
≥8	37	15		
Clinical Staging			13.25	0.002
≤T2a	14	18		
T2b	13	20		
≥T2c	49	34		
Age			2.89	0.090
<60	7	3		
≥60	69	69		
Biopsy of nerve invasion			23.86	<0.001
Yes	32	6		
No	44	66		
Prostate volume			4.72	0.095
<20	13	5		
20-59	54	55		
≥60	9	12		
Surgical approach			6.93	0.008
Robots	31	41		
Laparoscopy	45	31		

PSM, positive surgical margins; NSM, negative surgical margins.

We performed a multifactorial dichotomous logistic regression analysis with margin status (negative = 0, positive = 1) as the dependent variable and serum PSA level, Gleason score, percentage of positive puncture biopsy tissue, clinical stage, and surgical approach as independent variables. The results showed that perineural invasion, serum PSA, Gleason score, and percentage of positive stitches were independent risk factors for PSM (**Table 2**).

**Table 2:** Multi-factor dichotomous logistic regression analysis for the cut edge positive/cut edge negative group.

Variables	B	S.E.	Wald	df	sig.	Exp(B)	95% CI	
							Lower bound	Upper bound
Serum PSA	0.68	0.236	8.193	1	0.005	1.956	1.237	3.095
Percentage of positive stitches	0.504	0.230	4.837	1	0.029	1.653	1.059	2.588
Perineural invasion	0.851	0.427	4.109	1	0.044	2.358	1.031	5.407
Gleason Rating	0.482	0.206	5.436	1	0.03	1.624	1.10	2.429

### 3.2 Analysis of the risk factors for five-year survival

A univariate logistic regression analysis was performed, and after adjusting for stratification, the results showed that puncture biopsy for total prostate-specific antigen (tPSA), the nutritional status, lymph node metastasis, bone metastasis, and seminal vesicle invasion significantly affected the survival time of prostate cancer patients (p < 0.05). However, the other factors did not have a significant effect (p > 0.05) (**Table 3**).

**Table 3:** Comparison of univariate analysis of each risk factor for 5-year survival time.

Variables	PSM group (n=76)	NSM group (n=72)	*X^2^*	P value
Nutritional status			9.284	0.018
Poor	8	4		
Moderate	0	7		
Good	60	69		
BMI			0.462	0.415
Normal	46	59		
Abnormal	22	21		
Gleason score			0.281	0.527
5–6	2	4		
7–10	66	76		
Lymph node metastasis			5.847	0.006
No	46	36		
Yes	22	44		
Bone metastasis			24.477	<0.001
No	52	25		
Yes	16	55		
Seminal vesicle invasion			29.046	<0.001
No	28	10		
Yes	40	70		
Peripheral infiltration			2.162	0.060
No	53	51		
Yes	15	29		
Age			2.895	0.090
<60	7	3		
≥60	69	69		
PSA (ng/mL)			10.398	0.025
<10	4	15		
10-20	17	23		
≥20	55	34		

PSM, positive surgical margins; NSM, negative surgical margins; tPSA, total prostate-specific antigen; BMI, body mass index.

In the multivariate logistic regression equation, survival time was considered to be the dependent variable, and age stratification, puncture biopsy tPSA level, nutritional status, presence/absence of lymph node metastases, presence/absence of bone metastases, and presence/absence of seminal vesicle invasion were considered to be independent variables. The results showed that poor nutritional status and lymph node metastasis significantly affected survival time (p < 0.05) (**Table 4**).

**Table 4:** 5-year survival time multifactorial dichotomous logistic regression analysis.

Variables	B	S.E.	Wald	df	sig.	Exp(B)	95% CI	
							Lower bound	Upper bound
Intercept	−1.253	1.178	1.130	1	0.008	2.475	–	–
Nutrition = improper nutrition	1.720	0.846	4.136	1	0.032	5.583	1.064	29.285
Lymph = yes	−1.198	0.413	8.395	1	0.015	0.302	0.134	0.679

CI, confidence interval.

### 3.3 Logistic modeling and evaluation of PSM using ROC curves

**Figure 1:**
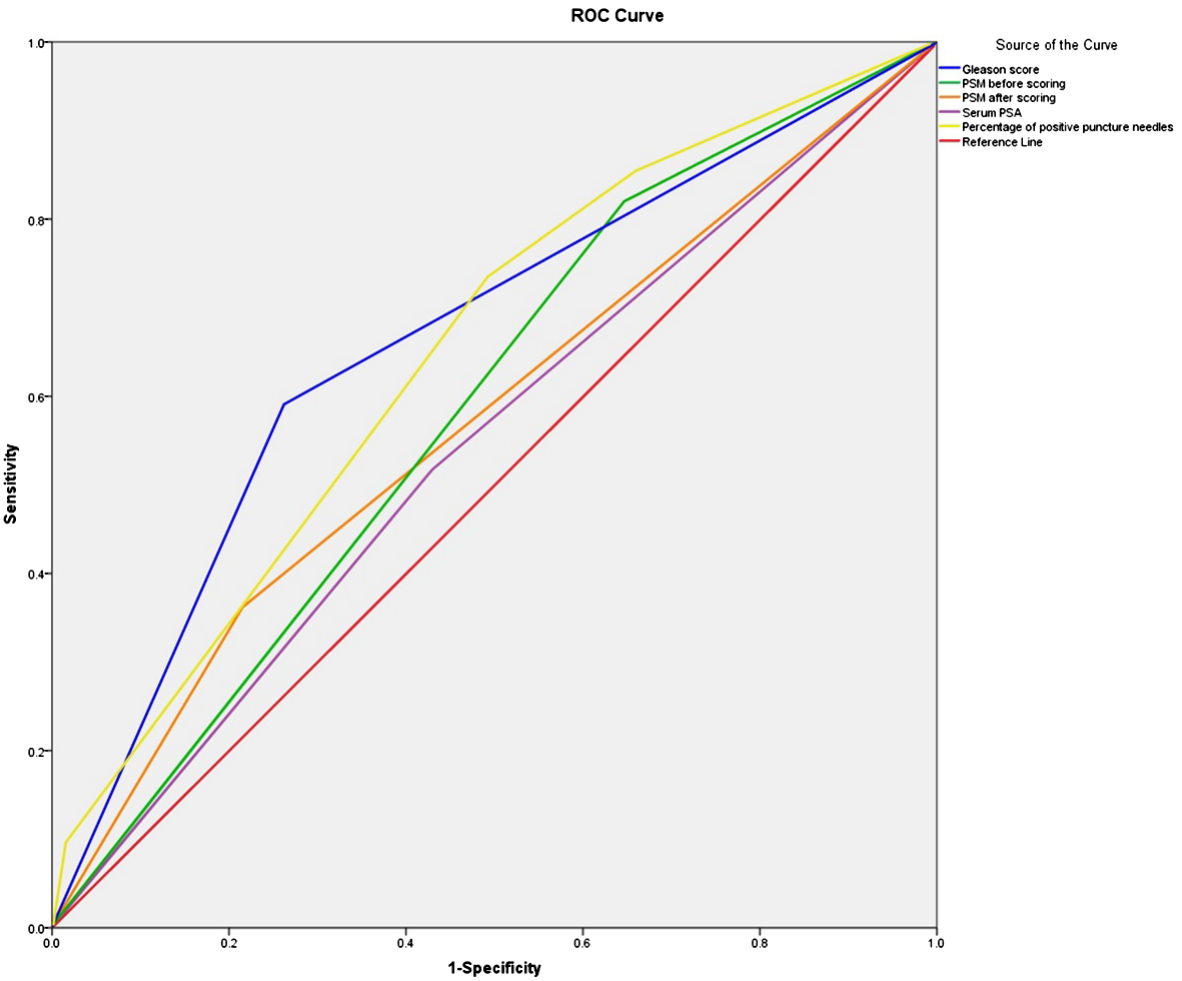
ROC curve of the survival time of PSM before and after scoring, Serum PSA, Percentage of positive puncture needles and Gleason score. ROC, receiver operating characteristic.

The area under the ROC curve before scoring was 0.787 (0.730–0.849), and the standard error was 0.029. The area under the ROC curve after scoring was 0.776 (0.725–0.854), and the standard error was 0.028. The difference in area between the two conditions was not significant, which indicated that the scoring also performed the same function. The scoring scheme showed moderate application validity. The Hosmer-Lemeshow test score was 4.929 (p > 0.05), suggesting a good fit. The area under the ROC curve for single risk factor prediction of PSM was 0.65 based on perineural invasion, 0.659 based on serum PSA, 0.728 based on the percentage of positive puncture needles, and 0.714 based on the Gleason score; all values were lower than the area under the ROC curve for the scoring system (**Table 5** and **Figure 1**).

**Table 5:** AUC of the ROC curve for evaluation of PSM with prostate cancer.

Test result variables	AUC	95% CI	
		Lower bound	Upper bound
PSM before scoring	0.787	0.730	0.849
PSM after scoring	0.776	0.725	0.854
Serum PSA	0.659	0.632	0.753
Percentage of positive puncture needles	0.728	0.692	0.804
Gleason score	0.714	0.675	0.754

AUC, area under the curve; ROC, receiver operating characteristic; CI, confidence interval.

### 3.4 Logistic modeling and prediction of patient survival time using ROC curves

**Figure 2:**
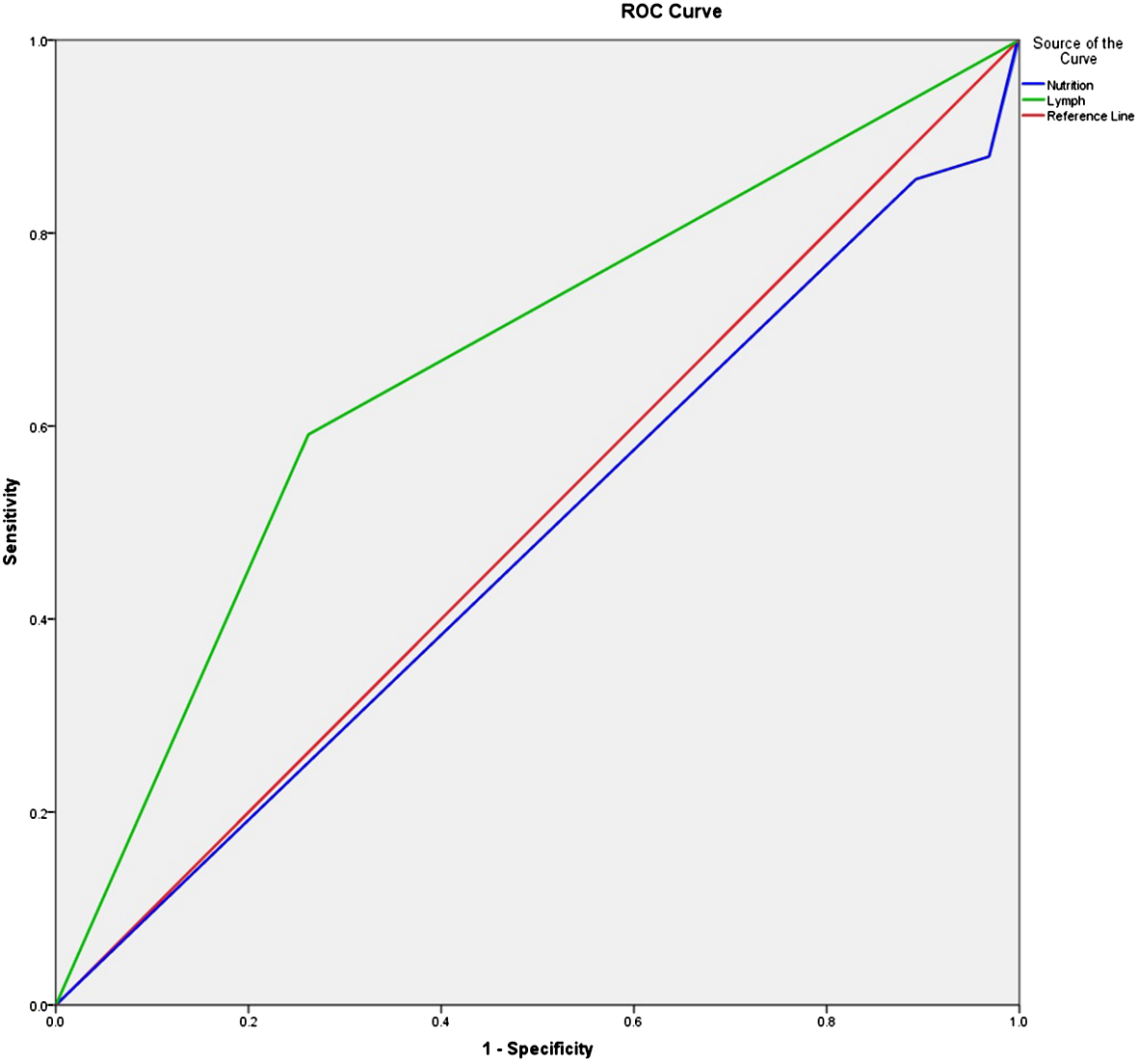
ROC curve of the survival time of prostate cancer patients receiving surgery. ROC, receiver operating characteristic.

Based on the results of the univariate and multivariate logistic regression analysis, we used two factors, i.e., poor nutritional status and lymph node metastasis, in the logistic model equation. The ROC curves were plotted using these two indicators as predictors and the composite predictor as the test variable. The AUC was calculated from these ROC curves, and the results showed that the AUC of lymph node metastasis (AUC: 0.664, 95% CI: 0.576–0.753) had a good predictive value, but poor nutritional status as a single predictive AUC did not have a clinically significant predictive value (AUC: 0.477, 95% CI: 0.384–0.571, P < 0.05) (**Table 6** and **Figure 2**).

**Table 6:** AUC of the ROC curve for predicting the survival time of patients with prostate cancer.

Test result variables	AUC	95% CI	
		Lower bound	Upper bound
Lymph	0.664	0.576	0.753
Nutrition	0.477	0.384	0.571

AUC, area under the curve; ROC, receiver operating characteristic; CI, confidence interval.

### 3.5 Determining the effectiveness of the PSM scoring system

A scoring system was established based on whether perineural invasion occurred following preoperative biopsy (yes = 2 points and no = 1 point), serum PSA (<10 ng/mL = 1 point, 10–20 ng/mL = 2 points, and >20 ng/mL = 4 points), Gleason score (<6 = 1 point, 7 = 2 points, and ≥8 = 4 points), and percentage of positive puncture stitches (<25% = 1 point, 25–49% = 2 points, and ≥ 50% = 4). A scoring system was established based on these parameters (minimum and maximum scores: 4 and 14, respectively). A risk score of 5.976 was associated with sensitivity and specificity of 66.7% and 80.9%, respectively. A risk score of 6.144 was associated with sensitivity and specificity of 66.3% and 84.4%, respectively. A risk score of 6.298 was associated with sensitivity and specificity of 59.5% and 86.6%, respectively. Based on the principle of optimal sensitivity and specificity, the highest sensitivity and specificity were achieved with a diagnostic score cut-off point of 6. Thus, the risk score cut-off point for PSM diagnosis was set at 6 (**Supplementary Table S1**).

Based on a cut-off value of 6, patients were divided into two groups: those at low risk of PSM (score 4–5) and those at high risk of PSM (score 6–14). Of the 148 patients included in the study, the proportion of patients in the PSM group with a score of 4–5 was 36.8% (28/76) and the proportion of patients in the PSM group with a score of 6–14 was 48.7% (37/76). By applying this scoring system to 50 patients admitted to our hospital with prostate cancer from January 2021 to January 2022, we detected nine PSMs and 31 negative cut-offs in the low-risk group, and eight PSMs and two negative cut-offs in the high-risk group (P = 0.001) (**Table 7**).

**Table 7:** Significance of PSM after prostate cancer resection in low and high risk groups.

Projects	PSM group	NSM group	Total	*χ*_2_	P value
Low risk 4-5 points	6	27	33		
High risk 6 - 14 points	6	1	7		
Total	12	28	40	11.985	0.001

PSM, positive surgical margins; NSM, negative surgical margins.

## 4 Discussion

Prostate cancer is a malignant tumor that occurs in prostate tissue. It results from the abnormal and disordered growth of prostate acinar cells (22). The disease generally has no specific symptoms in the early stage of onset. As the tumor grows, patients start showing symptoms such as frequent urination, urgent urination, slow urination, laborious urination, and even urine retention or urinary incontinence, which severely affects their quality of life (23, 24). Thus, patients with prostate cancer need to be treated with suitable methods for the best possible outcome. Clinicians are now paying greater attention to the quality of life of elderly patients with prostate cancer.

The most important indicator of complete tumor resection is whether the cut-edge status of the specimen is positive after prostate cancer resection. PSM is treated as a high-risk factor for disease progression after surgery, and urologists have investigated it for a long time. The presence of tumor cells at the cut edge of the specimen, based on a postoperative pathology report, indicates PSM, suggesting incomplete removal of the tumor. In a multicenter study, data were collected on the cut-edge status of 2,385 patients who underwent prostate cancer resection. Based on the National Comprehensive Cancer Network (NCCN) risk classification, the researchers found that the PSM rate was 19.1% (102/534) in the low-risk group, 26.0% (317/1218) in the moderate-risk group, 39.5% (153/387) in the high-risk group, and 81.8% (9/11) in the very high-risk disease group of patients (25). The results of this study were similar to the findings of the abovementioned study. Our findings also suggested that PSM after prostate cancer resection is strongly associated with postoperative biochemical recurrence and disease progression. A research group conducted a meta-analysis and found that PSM is associated with a high risk of biochemical recurrence after prostate cancer resection and may be an independent prognostic factor for the prognosis of patients with prostate cancer (26). Similarly, Jo et al. showed that after undergoing robot-assisted prostatectomy, PSM is a strong predictor of biochemical recurrence (27, 28). In the clinical setting, biochemical recurrence often indicates further disease progression, with the possibility of local recurrence or distant metastases, which can seriously threaten patient survival. Therefore, identifying the risk factors associated with PSM and establishing a scoring system for determining the risk of PSM after prostate cancer resection might provide a reference tool for preoperative prostate patients to predict the status of pathological margins after prostate cancer resection. Accurate predictions may help avoid PSM and improve patient prognosis.

In this study, we showed that PSM after prostate cancer surgery was associated with the post-penetration Gleason score, PSA level, seminal vesicle tumor infiltration, clinical stage of the tumor, size of the prostate volume, proficiency of the surgeon, the age and body mass index of the patient, biopsy nerve infiltration, and number of positive preoperative biopsy punctures (29, 30). Although these factors cannot be controlled, they suggest that early detection and treatment of prostate cancer is important, especially via PSA detection (8, 31–33). Therefore, for patients with prostate cancer receiving treatments via different methods, corresponding measures to improve the survival time need to be determined for better recovery of patients after surgery or follow-up radiotherapy and chemotherapy (34, 35). Illness-related behavior depends on the attitude and perspective of patients toward the disease; the mood, behavior, and level of cognition all influence the quality of life and well-being of patients (36, 37).The results of the binary logistic regression analysis showed that preoperative serum prostate-specific antigen, percentage of positive preoperative puncture stitches, puncture Gleason score, and preoperative puncture biopsy nerve invasion were independent risk factors for PSM. Tuliao et al. also found that the number of positive preoperative biopsy needles ≥3 (OR = 2.52, P = 0.043) was a predictor of postoperative PSM in a study on robot-assisted prostate cancer resection (38). Yang et al. retrospectively analyzed 296 patients who were diagnosed with rectal ultrasound prostate biopsy and treated with laparoscopic prostatectomy (OR = 4.403, 95% CI = 1.8). They found that the number of positive preoperative biopsy stitches (OR = 4.403, 95% CI = 1.878–10.325, P = 0.001) was an independent predictor of PSM (39). Heidenreich et al. performed a multifactorial analysis and found that a preoperative positive biopsy needle count of <50% was a significant predictor of pathologically negative margins after surgery for a clinically limited prostate cancer factor (40). In this study, we found that the percentage of patients with PSM was high (63.2%; 48/76) when the percentage of positive stitches was ≥50%. This makes the percentage of positive puncture stitches as the number of puncture stitches varies between institutions. In this study, the results of the univariate analysis showed that preoperative clinical staging was significantly different based on the margin status. The proportion of PSM patients with clinical staging ≥T2c was 64.5% (49/76), which was significantly higher than the proportion of PSM patients with clinical staging ≤T2b. Some researchers have found that clinical staging acts as an independent risk factor for PSM (41), but binary logistic regression analysis in this study showed that staging after clinical is not an independent risk factor for PSM. Koizumi et al. evaluated the clinical outcomes of 450 patients who underwent resective prostatectomy and conducted a multifactorial analysis. Their results showed that the mode of surgery was not an independent risk factor for PSM (42), which matches the results of this study.

In this study, a prospective case-control approach was used with strong causal inference. Based on the results of logistic regression, a quality-of-life prediction model with a good predictive effect on the survival time of patients can be established (43–46). Paying clinical attention to the observation and application of the abovementioned indicators can help predict the survival time of prostate cancer patients under treatment. Implementing relevant interventions early can be more effective in reducing the effect of risk factors; thus, improving the quality of life of patients (47–49). Evaluation of the clinical nutritional status, lymph node metastasis, and other indicators is suitable as additional costs and burdens are not imposed on patients for testing interventions (50, 51). Tools to evaluate disease-related behavior and family engagement support are also effective and suitable; moreover, they do not increase the burden on medical staff (52).

In this study, we identified the risk factors for PSM after prostate cancer resection. The area under the ROC curve was smaller than the area under the ROC curve of the scoring system if the PSM after prostate cancer resection was predicted by serum PSA, percentage of positive puncture stitches, the Gleason score, or perineural invasion alone. After determining the OR of each risk factor by binary multi-factor logistic regression analysis, scores were assigned, ROC curves were plotted, cut-off values were assessed, and finally, the risk prediction scoring system for PSM was established. The scoring system included the following risk factors: presence of perineural invasion on preoperative biopsy (yes = 2 points and no = 1 point), serum PSA (<10 ng/mL = 1 point, 10–20 ng/mL = 2 points, and >20 ng/mL = 4 points), Gleason score (<6 = 1 point, 7 = 2 points, and ≥8 = 4 points), and percentage of positive puncture stitches (<25% = 1 point, 25–49% = 2 points, and ≥ 50% = 4). Based on these parameters, we established a scoring system (minimum and maximum scores: 4 and 14, respectively). The clinical parameters of this scoring system were easily accessible, and the effectiveness of the scoring system was expressed by the area under the ROC curve. When the hazard score was 5.976, the sensitivity and specificity were 66.7% and 80.9%; when the hazard score was 6.144, the sensitivity and specificity were 66.3% and 84.4%; when the hazard score was 6.298, the sensitivity and specificity were 59.5% and 86.6%. For the best sensitivity and specificity, the hazard score cut-off value of this scoring system was found to be 6. We used a cut-off value of 6 to classify the patients into a “low-risk” group (4–5 points) with 36.8% PSM and a “high-risk” group (6–14 points) with 48.7% PSM. The differences between the risk groups were significant. While applying these factors as predictors of the scoring system, the combination of these factors showed that this scoring system is useful for the preoperative assessment of the clinical condition of patients.

This study had some limitations, for example, the operators were different, and postoperative pathology specimens were analyzed by different pathologists. Also, some indicators, such as body mass index, pathological staging, etc., were not included in the study. The sample size was too small to establish a scoring system. Thus, more patients need to be included, and a multicenter study needs to be conducted to validate our findings. As our study was descriptive, statistical analyses could not be performed to test any hypothesis.

## 5 Conclusions

To summarize, we identified several risk factors for PSM after prostate cancer resection. We found that serum PSA, percentage of positive puncture stitches, and the presence of perineural invasion by puncture are independent risk factors for PSM. A scoring system established based on these three factors showed moderate efficacy in predicting the risk of PSM after prostate cancer resection. For prostate cancer patients with a risk score below 6, the nerve can be preserved intraoperatively, whereas for those with a risk score of 6 and above, extended resection may be needed.

## Data Availability

The original contributions presented in the study are included in the article/**Supplementary Material**. Further inquiries can be directed to the corresponding author.

## Supplementary material

Supplementary Figure 1 Post-operative CT image of the patient’s prostate. Left, ADC; middle, DWI; right, T2.

Supplementary Table 1 Sensitivity and specificity of different diagnostic thresholds.
