# Corrigendum: Analysis of factors associated with positive surgical margins and the five-year survival rate after prostate cancer resection and predictive modeling

**DOI:** 10.3389/fonc.2024.1469673

**Published:** 2024-08-12

**Authors:** Kai Li, Yantao Zhang, Sinan Tian, Qingguo Su, Yanhui Mei, Wei Shi, Jingyuan Cao, Lijuan Song

**Affiliations:** ^1^ Department of Urology, Binzhou Medical University Hospital, Binzhou, China; ^2^ Department of Anesthesiology, Binzhou Medical University Hospital, Binzhou, China

**Keywords:** prostate cancer, radical prostatectomy (RP), survival time, logistic model, receiver operating characteristic curve (ROC curve)

In the published article, there was an error in affiliations 1 and 2. Instead of “Affiliated Hospital of Binzhou Medical College”, it should be “Binzhou Medical University Hospital”. The same affiliations have been updated in ***Methods, 2.1 General information***, paragraph 1.

In the published article, there was an error in the Ethics statement. The study was reviewed and approved by the Ethics Committee of the Affiliated Hospital of Binzhou Medical College (LW-99). The correct Ethics statement appears below and the same has been updated in the ***Methods, 2.1 General information***, paragraph 1.

