# Supplementary figures and images for: Analysis of factors associated with positive surgical margins and the five-year survival rate after prostate cancer resection and predictive modeling
Source: Front Oncol. 2024 Jun 6;14:1360404. doi: 10.3389/fonc.2024.1360404 (PMC11187091; doi:10.3389/fonc.2024.1360404)

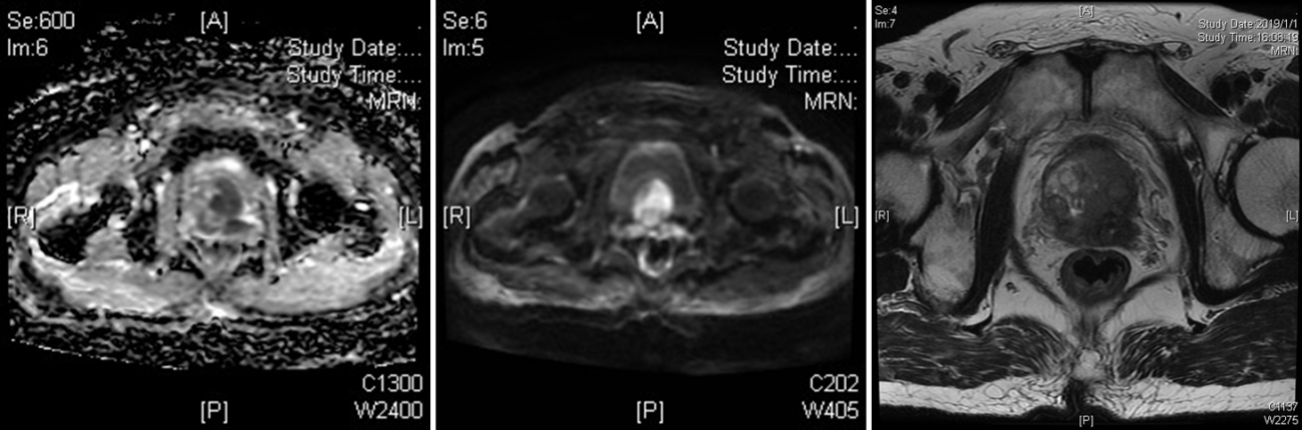

Supplement: Supplementary Figure 1 — Post-operative CT image of the patient’s prostate. Left, ADC; middle, DWI; right, T2. [file Image_1.tif]
