# Supplementary material for: Analysis of factors associated with positive surgical margins and the five-year survival rate after prostate cancer resection and predictive modeling
Source: Front Oncol. 2024 Jun 6;14:1360404. doi: 10.3389/fonc.2024.1360404 (PMC11187091; doi:10.3389/fonc.2024.1360404)
Supplement: Supplementary Table 1 — Sensitivity and specificity of different diagnostic thresholds. [file Table_1.docx]

**Table S1 Sensitivity and specificity of different diagnostic thresholds.**

| **Score** | **Sensitivity** | **Specificity** | **Sensitivity+Specificity** |
| --- | --- | --- | --- |
| 0.000 | 1 | 0.000 | 1.000 |
| 1.434 | 0.985 | 0.086 | 1.073 |
| 1.636 | 0.985 | 0.142 | 1.125 |
| 1.682 | 0.985 | 0.169 | 1.152 |
| 1.932 | 0.943 | 0.274 | 1.215 |
| 2.190 | 0.934 | 0.287 | 1.223 |
| 2.246 | 0.926 | 0.353 | 1.277 |
| 2.490 | 0.917 | 0.362 | 1.276 |
| 2.780 | 0.909 | 0.362 | 1.269 |
| 3.001 | 0.896 | 0.362 | 1.257 |
| 3.293 | 0.858 | 0.519 | 1.375 |
| 3.513 | 0.849 | 0.527 | 1.374 |
| 3.587 | 0.849 | 0.554 | 1.403 |
| 3.738 | 0.837 | 0.563 | 1.403 |
| 3.875 | 0.799 | 0.646 | 1.439 |
| 3.949 | 0.786 | 0.677 | 1.465 |
| 4.189 | 0.782 | 0.676 | 1.457 |
| 4.370 | 0.765 | 0.726 | 1.493 |
| 4.530 | 0.756 | 0.744 | 1.500 |
| 4.857 | 0.727 | 0.765 | 1.494 |
| 5.263 | 0.722 | 0.774 | 1.494 |
| 5.487 | 0.676 | 0.788 | 1.468 |
| 5.721 | 0.676 | 0.796 | 1.478 |
| 5.976 | 0.667 | 0.809 | 1.477 |
| 6.142 | 0.663 | 0.844 | 1.505 |
| 6.296 | 0.595 | 0.866 | 1.463 |
| 6.382 | 0.587 | 0.884 | 1.472 |
| 6.923 | 0.557 | 0.883 | 1.447 |
| 7.527 | 0.519 | 0.887 | 1.405 |
| 7.720 | 0.265 | 0.932 | 1.195 |
| 7.875 | 0.214 | 0.937 | 1.153 |
| 7.987 | 0.188 | 0.958 | 1.144 |
| 8.416 | 0.177 | 0.958 | 1.136 |
| 10.000 | 0 | 1 | 1.000 |
